# Management of patients at the hepatopancreatobiliary unit of a London teaching hospital during the COVID-19 pandemic

**DOI:** 10.1038/s41598-023-40264-9

**Published:** 2023-08-18

**Authors:** Sebastian M. Staubli, Dimitri A. Raptis, Shahi Ghani, Brian R. Davidson, Giuseppe K. Fusai, Charles Imber, Sateesh Iype, David Nasralla, Theodora Pissanou, Sakhawat Rahman, Dinesh Sharma, Pascale Tinguely, Fares Haddad, Miranda Dodd, Chris Dann, David Walker, Joerg-Matthias Pollok, On behalf of the Royal Free Hospital London HPB team, Abhirup Banerjee, Ahmed Mohamed, Aleem Obalogun, Alejandro Ramirez, Alex Rothnie, Andrej Grajn, Bhargava Chikkala, Bose Ojo-Williams, Camilla Hidalgo-Salinas, Carlo Ceresa, Carlo Frola, Charalampos Tsakiris, Conrad Shaw, Dimitrios Chasiotis, Elissaios Kontis, Farid Froghi, Guhan Venkatakrishnan, Helen Tzerbinis, Ioannis D. Kostakis, James Whitehead, Joao Costa, Krishnakumure Patel, Manikandan Kathirvel, Mitesh Sharma, Mohamed Elnagar, Murali Somasundaram, Nikolaos Dimitrokallis, Nolitha Morare, Nyasharenee Kupfuwa, Sandun Bulathsinhala, Shahroo Makhdoom, Stephanos Pericleous, Supreeth Kunnuru, Susana Ramos-Vazquez, Tameem Ibraheem, Gemma Keating, Linda Lightfoot, Sophie Brown, Lucy Gyamfi, Kajal Modi, Vanessa Tufuo, Harriet Louise Walker

**Affiliations:** 1 Department of HPB Surgery and Liver Transplantation, Clinical Service of HPB Surgery and Liver Transplantation, Royal Free London NHS Foundation Trust, UCL Partners, Pond Street, London, NW3 2QG UK; 2 Division of Surgery and Interventional Science, University College London, London, UK; 3 The Princess Grace Hospital (HCA Healthcare UK), London, UK; 4 Institute for Sport Exercise and Health (ISEH), University College Hospital London, London, UK; 5 Department of Trauma and Orthopaedics, University College London Hospitals NHS Foundation Trust, London, London, UK

**Keywords:** Viral infection, Liver diseases, Health services

## Abstract

To mitigate COVID-19-related shortage of treatment capacity, the hepatopancreatobiliary (HPB) unit of the Royal Free Hospital London (RFHL) transferred its practice to independent hospitals in Central London through the North Central London Cancer Alliance. The aim of this study was to critically assess this strategy and evaluate perioperative outcomes. Prospectively collected data were reviewed on all patients who were treated under the RFHL HPB unit in six hospitals between November 2020 and October 2021. A total of 1541 patients were included, as follows: 1246 (81%) at the RFHL, 41 (3%) at the Chase Farm Hospital, 23 (2%) at the Whittington Hospital, 207 (13%) at the Princess Grace Hospital, 12 (1%) at the Wellington Hospital and 12 (1%) at the Lister Hospital, Chelsea. Across all institutions, overall complication rate were 40%, major complication (Clavien–Dindo grade ≥ 3a) rate were 11% and mortality rates were 1.4%, respectively. In COVID-19-positive patients (n = 28), compared with negative patients, complication rate and mortality rates were increased tenfold. Outsourcing HPB patients, including their specialist care, to surrounding institutions was safe and ensured ongoing treatment with comparable outcomes among the institutions during the COVID-19 pandemic. Due to the lack of direct comparison with a non-pandemic cohort, these results can strictly only be applied within a pandemic setting.

## Introduction

The Royal Free London NHS Foundation Trust (RFHL) in London, United Kingdom (UK), is a major provider for treatment for benign and malignant hepatopancreaticobiliary (HPB) disease and liver transplantation (LT). Treatment of complex HPB and LT patients is resource intensive and requires specialised staff, organisation and infrastructure to ensure safe and efficacious treatment. During the COVID-19 pandemic, a large number of institutions re-allocated intensive therapy units (ITUs) and theatre capacity to treat patients with COVID-19, directly affecting the care for patients requiring HPB surgery^1–3^. The impact of the COVID-19 pandemic on healthcare providers through the absorption of treatment capacity and resources has previously been described as having led to delayed or insufficient treatment and threatened outcomes and patient safety, with increased morbidity and mortality rates^4–6^.

To counter the negative effects on patients requiring HPB treatment in institutions simultaneously carrying a high burden of COVID-19, a strategy to outsource patients with HPB pathology that included the respective treatment pathways, specialist staff and support services to surrounding hospitals potentially ensured the provision of ongoing specialist HPB care. Such a strategy carried inherent risks, as staff, expertise and equipment might not have been readily available in other hospitals. Furthermore, to the best of our knowledge, there are no data available regarding the safety and efficacy of outsourcing complex HPB patients ad hoc to other institutions.

To meet the ongoing demand for treatment of benign as well as malignant HPB disease, patients as well as surgical personnel (consultant surgeons, junior and senior clinical fellows/specialist registrars) were outsourced from the RFHL to surrounding NHS and private institutions. Patient treatment was aimed to be equal in all institutions. The patient outcomes were assessed and compared among the different hospitals, focusing on safety and efficiency. This study focuses on the surgical experience during the pandemic. We hypothesise that ensuring an integrated specialist team approach allowed specialist HPB surgical practise to continue when outsourced to the independent sector during the COVID-19 pandemic, avoiding delays in treatment while simultaneously ensuring safety and quality of care.

## Methods

This was a prospective audit with data collected from November 2020 until October 2021 of all consecutive patients who underwent treatment under the HPB unit at the Royal Free Hospital (RFHL) in one of the following six hospitals in Greater London, UK: RFHL, Chase Farm Hospital (CFH), Whittington Hospital (WH), Wellington Hospital North (WHN), Princess Grace Hospital (PGH) and Lister Hospital, Chelsea (LIS), three of which are NHS institutions (RFHL, CFH, WH), and three being private hospitals (PGH, WHN, LIS). PGH and WHN provided an environment with already established HPB practices under five consultants. This audit was approved by the institution’s quality governance department, audit and compliance (Royal Free Hospital, London, UK). Anonymised data collection was prospectively performed via electronic patient records by the perioperative team at the time of discharge. This audit was registered by the Royal Free London audit tracker under the identifier RFH 662_22/23. As per UK Policy Framework for Health and Social Care Research, no ethics approval or informed consent was needed due to the nature of this quality improvement clinical audit project (COREC: ethics consultation e-group Audit, research or service evaluation, Oct 05)^7^. The authors confirm that all methods were carried out in accordance with the relevant guidelines and regulations.

### Patient management

During the COVID-19 pandemic, patients suffering from HPB cancers were discussed at a weekly meeting consisting of the surgeons, anaesthetists and administrative and managerial personnel of the above-mentioned institutions. Primary criteria for case allocation to surrounding institutions were availability of ITUs and theatre capacity and were in accordance with the representatives of the respective institutions. Surgical treatment for malignant disease was allocated to RFHL or PGH, depending on the availability of advanced surgical and ITU capacity.

Operations for benign disease were performed in case of emergency or severe symptoms. In principle, these cases were attributable to all institutions. Complex surgery for benign disease requiring postoperative ITU treatment was performed in RFHL and PGH. Medical criteria for case allocation for cholecystectomies were defined as medically and surgically non-complex (CFH), medically complex and surgically non-complex (WH).

COVID-19 screening policy was identical across all mentioned institutions. Patients who underwent treatment had to provide a negative COVID-19 lateral flow test (LFT) 72 h before admission and self-isolate for 14 days prior to admission. In case of occurring symptoms, immediate re-testing (LFT) was required. Patients were admitted 1 day preoperatively, tested for COVID-19 (PCR) and and handed out a screening questionnaire to identify those at risk for COVID-19. Hospital staff were asked to regularly self-test (LFT) on a weekly basis or if symptoms occurred.

A rota for the HPB team was created that included coverage of the above-named institutions. One workforce did not cover more than one institution simultaneously, and total amount of workhours and working days per week remained unchanged. Surgical coverage included surgical treatment, perioperative management, ward coverage and on-calls and was provided by consultants, senior clinical fellows and junior clinical fellows from the RFHL. The number of staff from the RFHL remained unchanged, as elective work and benign clinic work had halted, freeing up capacity for surrounding institutions.

Anaesthetic staff were provided by the RFHL and the Royal London Hospital in case of staff shortage. An anaesthetist involved in an operation would remain on call for their patient until 0700 A.M. the next morning, in case the patient required urgent re-operation. Anaesthesiologic on-call coverage over the weekends was provided by private anaesthesiologists.

Twenty-four-hour cover for interventional radiology (IR) and endoscopy services were available at the RFHL. In the private institutions, no on-call systems for IR and endoscopy were available, and surgical consultants had to contact individual specialists in emergency cases. No out-of-hours resident theatre staff were available in the private institutions, but on-call coverage was provided from home.

To ensure consistent quality of treatment, treatment pathways including postoperative management, enhanced recovery after surgery, intermediate care/high dependency unit (HDU) and ITU treatment plans as well as ITU step-down plan criteria were conceptualised. Patients who underwent laparotomy were managed postoperatively in an HDU or ITU based on medical complexity. The remaining cases went postoperatively straight to ward, HDU or ITU, based on medical complexity and comorbidities.

### Patient characteristics

All consecutive patients who underwent treatment under the HPB and liver transplant service at the RFHL between November 2020 and October 2021 in the RFHL and its affiliated institutions were included in this audit. Benign as well as malignant disease were included in the analysis. All tumour types from the following organ systems were registered: liver, gallbladder, pancreas, biliary and sarcoma. Patients undergoing liver transplantation for all indications were included. Patients requiring complex interventional treatment or peri-interventional hospitalisation were admitted under the HPB unit and included in the analysis. Operations for benign conditions were performed in emergency cases.

Patients with complications after routine day-surgery endoscopy or after elective radiological interventions with mandatory 24-h stay were admitted under the HPB unit and included in the cohort. No systematic follow-up was conducted after discharge of the patients. Data of patients readmitted to the RFHL were captured.

### Variables

Variables used were sex, age, affected organ system of the main treated diagnosis, benign or malignant disease, comorbidity scores classified according to the Charlson Comorbidity Index (CCI®)^8^, patient location (hospital) and postoperative complications as measured with the Clavien–Dindo Classification^9,10^ and Comprehensive Complications Index® (CCI®)^11^. Treatment characteristics included operation, intervention or medical/conservative management, resectability of cancer as well as abandoned resections.

Grouping of patients for analysis was conducted according to the main affected organ relevant for treatment. Cholangiocarcinoma (CCC) cases were subsumed under the organ category ‘liver’ if concomitant liver resection was performed. Cases of distal CCC with concomitant pancreatic resection were subsumed under the organ category ‘pancreas’.

### Main outcomes

The main outcomes of this study were the total number of patients treated operatively in each centre and the short-term outcomes in these patients. Patients suffering from malignancy were analysed separately. Short-term postoperative outcomes included length of hospital stay, overall major morbidity and mortality until hospital discharge for all cases. Major morbidity was defined as a complication grade 3a or higher according the Clavien–Dindo classification until discharge^9^. Mortality was defined as death during hospitalisation in the primary admission. Patients developing COVID-19 during the hospitalisation were captured, and their outcomes analysed separately. Patients were grouped in the analysis by hospital, namely RFHL and affiliated recipient institutions, as malignant and benign as well as per type of intervention (conservative, intervention or operation). The sample size was defined as the total number of patients treated in a 1-year period during the COVID-19 pandemic between 01 Nov 2020 and 30 Oct 2021.

Surgical case complexity was measured for major liver and pancreatic surgery. For liver surgery, complexity of the procedure was measured by adding points in case one or several of the following parameters were present: Hilar lymphadenectomy, multiple resections in one procedure, additional ablation to resection, venous resection, biliary reconstruction, and concomitant enteric resection^12^. For pancreatic surgery, complexity of procedure was measured by screening for the following parameters: BMI over 35 kg/m^2^, soft pancreas, small duct (< 3 mm), distal pancreatectomy, total pancreatectomy, venous reconstruction, arterial reconstruction, and extended resection^13^. For each presented item one point was added, and a final complexity score for each procedure was measured.

Medical case complexity was defined as comorbidities (CCI®), caputured for each case during data acquisition^8^.

To prevent confirmation bias, data analysis was performed after complete data acquisition.

### Statistical methods

Continuous data were reported as mean and standard deviation (SD) or median and inter-quartile range (IQR), where appropriate. Categorical data were reported as frequencies (n) and proportions (%). Continuous variables were compared with the Student’s t, Mann–Whitney U, one-way ANOVA and Kruskal–Wallis tests, where appropriate. Differences among proportions derived from categorical data were compared using the Fischer’s exact or the Pearson χ^2^ tests, where appropriate. All p values were two-sided and considered statistically significant if *p* ≤ 0.05. Statistical analysis was performed using R Studio version 1.0.44 (RStudio, Inc. GNU Affero General Public License v3, Boston, MA, 2016) with the rBiostatistics.com beta version graphical user interface (rBiostatistics.com. Cloud Graphical User Interface for R Statistics and eLearning Platform. London, UK, 2017)^14^.

## Results

**Figure 1:**
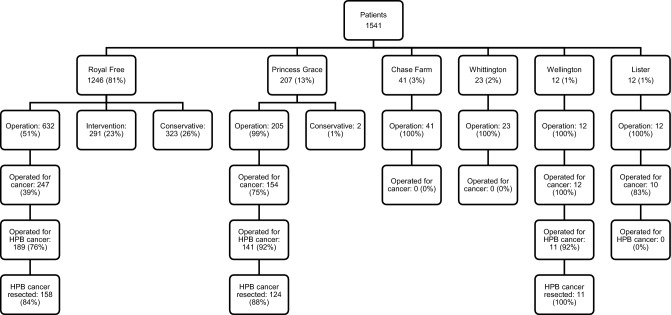
Overview of patients treated stratified by centre, type of treatment and cancer operation.

Between November 2020 and October 2021, 1541 patients were included in the analysis. Grouped by centre, 1246 (80.8%) were treated at RFHL, 41 (2.7%) at CFH, 23 (1.5%) at WH, 207 (13.4%) at PGH, 12 (0.8%) at WHN and 12 (0.8%) at LIS, as shown in Fig. 1. There were no missing data in all measured outcome parameters.

### Patient characteristics

Patient, disease and treatment characteristics of patients treated in this setting are shown in Table 1. Across all institutions, 60% (925/1541) patients were treated operatively, 18.9% (291/1541) underwent an intervention and 21.1% (325/1541) underwent conservative treatment. Grouped by organ system, and including all treatment types, a majority of the patients were treated for liver (23.7%; 347/1541), pancreas (24.8%; 362/1541) and gallbladder (26.1%, 283/1541) disease. Median CCI® in the entire cohort was 3 (IQR 1–5) and 5 (IQR 1–5) in patients who underwent surgery for malignancy.

**Table 1:** Baseline patient characteristics.

Characteristic	n = 1541	
Age group		
0–24	1	0.95%
25–49	298	20.26%
50–59	308	20.94%
60–69	352	23.93%
70–79	331	22.50%
> 80	168	11.42%
Mean age	59.49 (SD 15.59)	
Sex		
Female	704	46.1%
Male	820	53.8%
Hospital		
Chase Farm Hospital	41	2.7%
Lister Hospital	12	0.8%
Princess Grace Hospital	207	13.4%
Royal Free Hospital	1246	80.8%
Whittington Hospital	23	1.5%
Wellington Hospital North	12	0.8%
Treatment type		
Conservative	325	21.1%
Intervention	291	18.9%
Operation	925	60%
Organ group		
Biliary	114	7.8%
Gallbladder	283	26.1%
Hernia	27	1.8%
Liver	347	23.7%
OLT	94	6.4%
Other	65	4.4%
Pancreas	362	24.8%
Sarcoma	63	4.3%
Spleen	4	0.3%
*UGI*	4	0.3%
Number of comorbidities		
Minimum	0	
Maximum	16	
Median	3	
IQR (25–75%)	1–5	
CCI® Index		
Minimum	0	
Maximum	100	
Median	0	
IQR (25–75%)	0–8.7	

### Perioperative outcomes

Total numbers of patients as well as cancer operations, grouped by organ system including liver transplants for cancer, are subsumed in Table 2. A total of 184 patients were treated surgically for cancer necessitating a liver resection. Some 95% of these cases (176/184) were deemed primarily resectable; of these, 95% (167/176) were resected, and in nine cases (5%), resection was abandoned. A total of 158 patients were treated surgically, with their cancer necessitating pancreatic resection. Some 96% (152/158) of these cases were deemed primarily resectable; of these, 82% (125/152) were resected, and in 27 cases (18%), resection was abandoned.

**Table 2:** Surgical procedures.

Characteristic		
All operations (per disease group)		
n = 925		
Gallbladder	289	31%
Liver	225	24%
Pancreas	175	19%
OLT	87	9%
Sarcoma	65	7%
Other	32	3%
Hernia	28	3%
Biliary	17	2%
UGI	4	1%
Spleen	3	1%
Cancer operations (per disease group)		
n = 423		
Liver	184	43%
Pancreas	158	37%
Sarcoma	64	15%
OLT	14	14%
Biliary	3	1%

Outcomes and complications after surgical, interventional and conservative treatment of patients admitted under the HPB unit across all institutions are summarised in Table 3. The complication rate in patients treated operatively across all institutions was 40% (368 patients), with a major complication rate of 11% (105 patients) and a mortality rate of 1.4%. In patients treated interventionally, the complication rate was 27% (77 patients) and the major complication rate was 9% (27).

**Table 3:** Complication rates across all institutions.

Characteristic		
Highest complication grade		
None	1092	70.9%
1	85	5.6%
2	228	14.8%
3a	33	2.9%
3b	32	2.1%
4a	27	1.8%
4b	13	0.8%
5	20	1.3%
Complication of any severity	449	29.1%
Major Complication (Grade > 2)	135	8.8%
In-hospital death	135	1.4%

In all operated patients, grouped by organ system, the total number of complications, major complications and mortalities for liver (n = 225) was 111 (49%), 29 (13%) and 2 (0.9%), respectively. For pancreatic surgery (n = 176), the overall complication rate was 112 (64%), the major complication rate was 27 (15%) and 4 (2.3%) mortalities ensued. For gallbladder surgery (n = 289), 31 (11%) complications were recorded, of which were 6 (2%) major complications, in the absence of fatal outcomes. For sarcoma surgery (n = 65), 21 (32%) complications ocurred, with 5 (8%) major complications and no mortalities. For OLT (n = 87), the overall complication was 65 (75%), and the major complication rate was 28 (32%).A total of 4 (4.6%) mortalities were recorded.

### Outcomes in COVID-19-positive patients

A total of 28 patients (2%) were COVID-19 positive in the reported cohort. The complication rate in COVID-19-positive patients was increased, compared with the general operated patient population, as shown in Table 4. Patients in this cohort who suffered from COVID-19 were diagnosed during the hospitalisation. The diagnosis was confirmed with PCR tests in all cases.

**Table 4:** Covid-19-related outcomes.

	Covid-19 −	Covid-19 +	Univariate analysis	
			OR (95% CI)	*p*-value
n	925	28		
Overall complication rate (%)	42	72	4 (1–13)	0.015
Major complication rate (%)	10	44	7 (2–20)	< 0.001
Mortality (%)	1	11	12 (1–72)	0.022

OR, odds ratio; 95% CI, 95% confidence interval.

### Comparison between public (NHS) and private institutions

Mean (sd) case complexity scores of major liver operations stratified by public (NHS) and private institutions were 0.45 (0.67), and 0.48 (0.60) (*p* = 0.751), respectively. Mean (sd) case complexity scores for pancreatic operations were 0.71 (0.54), and 0.87 (0.45) (*p* = 0.038), respectively. Medical case complexity (CCI®) scores between liver and pancreas surgical groups in public (NHS) and private institutions showed no significant differences. In the liver group, mean (sd) CCI® scores for patients operated in the public (NHS) and private institutions were 5.29 (3.29), and 5.56 (3.07) (*p* = 0.545), respectively. For pancreatic surgery, mean (sd) CCI ® scores were 4.48 (2.38), and 4.57 (2.33) (*p* = 0.82), respectively.

The outcomes of the institutions contributing the largest number of patients to this study were compared to assess whether the outcomes were similar. The RFHL and PGH contributed a total of 1246 and 207 patients, respectively. In the RFHL, 632 patients were operated, of whom 247 were for cancer and 189 for HPB cancer. Of these, 182 were seen as potentially resectable and 158 were resectable. In PGH, 205 patients were operated, of whom 154 were for cancer and 141 for HPB cancer. Of these, 135 were seen as potentially resectable and 124 were resectable. Comparing the complication rates in patients with resection for cancer in both hospitals, the overall number of complications was 99 (63%) in the RFHL and 77 (62%) in PGH. The number of major complications was 24 (15%) in the RFHL and 12 (10%) in PGH (*p* = 0.673).

## Discussion

The main finding of this report is that ad hoc outsourcing of patients with complex HPB disease, as well as of a full complement of senior and junior surgical teams, during the COVID-19 pandemic was safe and feasible. This approach offers a possible workaround solution for other hospitals should a pandemic-related shortage of treatment capacity arise in the future.

The total numbers of treated patients showed a relatively large difference between the two main hospitals, with a ratio of approximately six to one, giving the impression of a small contribution by the external sector. However, the numbers are much more equilibrated when the subgroup of cancer patients are compared, with 247 and 154 patients operated at the RFHL and the PGH, respectively. Further subdivision of the cohort of HPB cancer resection leads to even more balanced numbers of 158 and 124 patients in the RFHL and PGH, respectively. During the COVID-19 surge, treatment capacity for patients requiring complex HPB surgery at the RFHL was scarce, and these cancer patients would have been likely to have suffered critical treatment delays or not received adequate treatment in time. The PGH acted as an overflow basin in this situation, and patients would have been likely to have suffered substantial treatment delays with increased morbidity and cancer-related mortality had this additional treatment capacity not been available^15,16^. Of note, patients were allocated to the respective institutions as per the availability of capacity and did not follow guidelines or other set criteria. In the future, a set of criteria for patient allocation needs to be defined to improve this process. An alternative way to read our data would be that a relatively large number of patients were treated safely at the RFHL amid pandemic conditions. As ITU occupancy with COVID-19 was high and available beds for major surgery were limited, outsourcing of cases allowed for the ongoing provision of specialist HPB care.

In this study, we have reported on our experience during the COVID-19 pandemic while aiming to provide uninterrupted, ongoing care of patients suffering from HPB malignancy while maintaining a caseload comparable with pre-pandemic conditions. Therefore, surgical training of junior staff as well as consultants’ skill maintenance could be ensured due to them having a case load consistent with the pre-pandemic levels. Most operations performed at the PGH were cases led by consultants. This affected training during this period, but exposure to complex surgical procedures was still offered to trainees, compared with institutions that had brought surgical activity to a complete halt. Furthermore, ongoing provision of HPB treatment enabled the prevention of an increase in the waiting list backlog, which potentially affected many institutions throughout the UK, with poorly understood long-term consequences.

Due to the completeness of the presented data, this pathway could be critically assessed. Our data showed that outsourcing patients from a tertiary HPB centre to surrounding institutions was feasible and safe, not least because of the provision of specialised personnel to the recipient institutions. Providing necessary staff is vital, as a shortage of trained personnel would be likely to present a bottleneck in this situation. The safety of this approach is further underlined when comparing our outcomes against respective benchmark values^17,18^. In this study, the CCI® was calculated for every individual patient. This index represents the sum of complications and is weighted for their severity. In our cohort, the CCI® benchmark values were not breached. This is especially interesting, as these benchmark values were calculated under non-pandemic conditions, and the influence of the COVID-19 pandemic on patient outcomes is not yet fully understood. Direct comparison of surgical case complexity between NHS and private institutions showed no statistically significant difference for liver surgery, however, pancreatic procedure complexity was significantly higher in private institutions. The clinical significance of this result is debatable, as outcomes were comparable in both major institutions (RFHL and PGH). Medical case complexity was not significantly different in both public and private institutions, allowing the conclusion that the cases were comparable from a surgical and medical complexity viewpoint.

Of note, information technology (IT) systems were not compatible between the main institutions (RFHL and PGH), and loss of electronic patient data and PACS datasets was frequently encountered, requiring the additional efforts of staff involved to counteract this weakness. The same effect was encountered in the opposite direction, when patients who received initial treatment at another institution were followed up at the RFHL. In the future, standardisation of IT systems or patients holding an electronic copy of their personnel records could solve this issue.

Another relevant limitation of this study is the lack of systematic follow-up of patients. To gain further insight into long-term surgical outcomes, 90-day follow-up data would be needed, but only the short-term outcomes during hospitalisation were available. Due to the lack of this data, long term oncological outcome data cannot be presented in this paper. In this prospective audit, care needs to be taken when comparing outcomes between different institutions, as case complexity was not prospectively recorded. Even though patients were primarily allocated to surrounding institutions according to the availability of surgical as well as intensive care treatment capacity, a bias towards higher risk patients at the tertiary HPB centre is possible. This is especially true for patients with central CCC, who were operated solely at the RFH. However, those cases represented a minority of patients, and apart from those cases, patients were allocated according to the available treatment capacity. Furthermore, this study exclusively covers patient outcomes and does not offer any information regarding the additional stress on healthcare professionals of working in different environment and hospitals. Of note, this is not primarily a comparative analysis of different centers and hospitals, and a possibility of selection bias for each center remains. In this paper, no comparison of the outcomes during the pandemic with pre- or post-pandemic conditions is presented, which represents a limitation of this work.

To further improve the above-mentioned system, several issues need to be addressed. These include refinement of patient selection for outsourcing and identification of patient characteristics that predict favourable outcomes in patients outsourced to different institutions. To further refine the case allocation process, specialist meetings in each tumour group to define appropriate cases should be implemented in the future. A further relevant point that needs to be addressed is that private institutions did not provide endoscopy or IR services out of hours. In emergency cases, the respective specialists had to be contacted individually, which caused treatment delays. Similarly, these hospitals did not provide resident our-of-hours theatre staff. Patients returning to theatre required staff to come from home, causing treatment delays. These issues can be overcome, but they represent major gaps and could even lead to difficulties with critical incidents.

Furthermore, an ad hoc outsourcing system could potentially lead to increased stress for the outsourced staff as well as the staff at the recipient hospital, who might not be used to the workload or complexity of the new patients. Identifying the main stressors as well as strategies to relieve stress for staff in this context would be a rewarding topic to address, as optimisation of the work environment could improve patient outcomes and staff well-being, despite an ongoing pandemic.

Specialised pathways for surgical inpatient treatment have been described, with a focus on the avoidance of perioperative COVID-19 infection^19,20^. These attempts to create a safe non-COVID-19 treatment area, however, often came at the expense of an overall reduction in numbers of treated patients^21^ and were mainly practiced during the first COVID-19 wave. While these efforts were undoubtedly of crucial relevance, they did not solve the issue of overall reduction of treatment capacity. Our approach to outsource patients to other institutions therefore did not compete but rather complemented the above-mentioned pathways.

Overall, evidence of the efficacy and safety of treatment pathways during the COVID-19 pandemic is slowly emerging, but there is a need for further critical assessments of the management of non-COVID-19 patients during the pandemic. To the best of our knowledge, this is the only study that specifically addresses a population of patients under HPB care during the COVID-19 pandemic.

Since the authors of this paper would like to offer a possible solution for treatment shortage for other HPB institutions in a pandemic situation in the future, the generalisability of the management of the proposed approach is of vital importance. To allow other institutions to adopt a similar system, the availability of surrounding treatment capacity is necessary, including ITU capacity. Therefore, the described approach is especially helpful in a healthcare system that centralises patients suffering from COVID-19, thereby freeing ITU capacity in other hospitals that then can accept other complex patients in return. Furthermore, organising such a system requires significant managerial efforts, and the allocation of resources to this task is necessary. To be able to develop contingency plans in the future, there is a need for further reporting of other institutions with outsourcing of specialist services and comparisons to enable the identification of factors that are essential to successful outsourcing for specialist services.

In conclusion, outsourcing of patients requiring complex HPB treatment and surgical staff from a tertiary centre to smaller surrounding institutions can be a viable solution to ensure ongoing patient treatment in a pandemic situation. A sufficient body of experienced surgeons, additional organisational efforts and close inter-institutional communication are a prerequisite for success in such a setting.

## Data Availability

Authors agree to make data and materials supporting the results or analyses presented in their paper available upon reasonable request. Please contact the corresponding author.
