# Author Correction: Management of patients at the hepatopancreatobiliary unit of a London teaching hospital during the COVID-19 pandemic

**DOI:** 10.1038/s41598-023-44594-6

**Published:** 2023-10-16

**Authors:** Sebastian M. Staubli, Dimitri A. Raptis, Shahi Ghani, Brian R. Davidson, Giuseppe K. Fusai, Charles Imber, Sateesh Iype, David Nasralla, Theodora Pissanou, Sakhawat Rahman, Dinesh Sharma, Pascale Tinguely, Fares Haddad, Miranda Dodd, Chris Dann, David Walker, Joerg-Matthias Pollok, On behalf of the Royal Free Hospital London HPB team, Abhirup Banerjee, Ahmed Mohamed, Aleem Obalogun, Alejandro Ramirez, Alex Rothnie, Andrej Grajn, Bhargava Chikkala, Bose Ojo-Williams, Camilla Hidalgo-Salinas, Carlo Ceresa, Carlo Frola, Charalampos Tsakiris, Conrad Shaw, Dimitrios Chasiotis, Elissaios Kontis, Farid Froghi, Guhan Venkatakrishnan, Helen Tzerbinis, Ioannis D. Kostakis, James Whitehead, Joao Costa, Krishnakumure Patel, Manikandan Kathirvel, Mitesh Sharma, Mohamed Elnagar, Murali Somasundaram, Nikolaos Dimitrokallis, Nolitha Morare, Nyasharenee Kupfuwa, Sandun Bulathsinhala, Shahroo Makhdoom, Stephanos Pericleous, Supreeth Kunnuru, Susana Ramos-Vazquez, Tameem Ibraheem, Gemma Keating, Linda Lightfoot, Sophie Brown, Lucy Gyamfi, Kajal Modi, Vanessa Tufuo, Harriet Louise Walker

**Affiliations:** 1 Department of HPB Surgery and Liver Transplantation, Clinical Service of HPB Surgery and Liver Transplantation, Royal Free London NHS Foundation Trust, UCL Partners, Pond Street, London, NW3 2QG UK; 2 Division of Surgery and Interventional Science, University College London, London, UK; 3 The Princess Grace Hospital (HCA Healthcare UK), London, UK; 4 Institute for Sport Exercise and Health (ISEH), University College Hospital London, London, UK; 5 Department of Trauma and Orthopaedics, University College London Hospitals NHS Foundation Trust, London, London, UK

Correction to: *Scientific Reports* 10.1038/s41598-023-40264-9, published online 18 August 2023

The original version of this Article contained an error in the spelling of the author Harriet Louise Walker which was incorrectly given as Louise Walker Harriet. She is a member of the consortium “On behalf of the Royal Free Hospital London HPB team”.

The original Article has been corrected.

